# Eye Movement Measures of Within-Language and Cross-Language Activation During Reading in Monolingual and Bilingual Children and Adults: A Focus on Neighborhood Density Effects

**DOI:** 10.3389/fpsyg.2021.674007

**Published:** 2021-10-27

**Authors:** Veronica Whitford, Marc F. Joanisse

**Affiliations:** ^1^Department of Psychology, University of New Brunswick, Fredericton, NB, Canada; ^2^Department of Psychology, Brain and Mind Institute, Western University, London, ON, Canada

**Keywords:** bilingualism, monolingualism, reading, eye movements, within-language and cross-language activation, orthographic neighborhood density, children, young adults

## Abstract

We used eye movement measures of first-language (L1) and second-language (L2) paragraph reading to investigate how the activation of multiple lexical candidates, both within and across languages, influences visual word recognition in four different age and language groups: (1) monolingual children; (2) monolingual young adults; (3) bilingual children; and (4) bilingual young adults. More specifically, we focused on within-language and cross-language orthographic neighborhood density effects, while controlling for the potentially confounding effects of orthographic neighborhood frequency. We found facilitatory within-language orthographic neighborhood density effects (i.e., words were easier to process when they had many vs. few orthographic neighbors, evidenced by shorter fixation durations) across the L1 and L2, with larger effects in children vs. adults (especially the bilingual ones) during L1 reading. Similarly, we found facilitatory cross-language neighborhood density effects across the L1 and L2, with no modulatory influence of age or language group. Taken together, our findings suggest that word recognition benefits from the simultaneous activation of visually similar word forms during naturalistic reading, with some evidence of larger effects in children and particularly those whose words may have differentially lower baseline activation levels and/or weaker links between word-related information due to divided language exposure: bilinguals.

## Introduction

Though seemingly effortless, visual word recognition is a complex process that involves accessing and retrieving correct lexical representations from the mental lexicon, often among a pool of visually similar lexical candidates known as orthographic neighbors. The classic definition of orthographic neighbors includes substitution neighbors: words that resemble a target word in all but one letter, regardless of that letter’s position in the target word (Coltheart et al., 1977). For example, the English word *horse* has the following within-language (English) substitution neighbors: *horde*, *house*, *horst*, *horsy*, *Morse*, *Norse*, and *worse*. However, an updated definition also includes addition and deletion neighbors: neighbors with one additional or one fewer letter (Davis et al., 2009). For example, the English word *horse* has the following within-language (English) addition: *hoarse*, *horses*, and *horsey* and deletion: *hors* and *hose* neighbors. Alongside within-language neighbors, words can also have cross-language neighbors. For example, the English word *horse* has the following cross-language (French) substitution: *corse*, *horde*, *morse*, and *torse* and deletion: *hors* neighbors.

Whether within-language or cross-language, a target word’s total number of orthographic neighbors (substitution + addition + deletion) is called its orthographic neighborhood density, and the average word frequency of its orthographic neighbors is called its orthographic neighborhood frequency. Although both properties can exert robust influences on visual word recognition, the extant research has predominantly focused on monolingual young adults, which may lack generalizability to other populations. The current study aims to address this imbalance in the literature by investigating both within-language and cross-language orthographic neighborhood density effects (while controlling for orthographic neighborhood frequency) during naturalistic paragraph reading in four participant groups differing in age and language background: (1) monolingual children; (2) monolingual young adults; (3) bilingual children; and (4) bilingual young adults. We begin with an overview of what is known about orthographic neighborhood effects among monolingual children and young adults, followed by that among bilingual children and young adults.

### Monolingual Orthographic Neighborhood Effects

#### Theoretical Framework

Leading theories of monolingual visual word recognition, such as the Interactive Activation (IA) model (McClelland and Rumelhart, 1981), propose that the activation of many orthographic neighbors (i.e., a high orthographic neighborhood density) impedes lexical access of a target word. This is especially true for higher-frequency orthographic neighbors (i.e., a high orthographic neighborhood frequency), which have higher baseline activation levels and/or higher quality lexical representations (see Perfetti, 2007 for a discussion of the lexical quality hypothesis). This impedance is attributed to lateral inhibition. As orthographic units (i.e., letters and their clusters, such as bigrams) are identified, multiple lexical candidates containing these orthographic units compete for activation, especially higher-frequency candidates; they send the most lateral inhibition. Lower-frequency candidates, which have lower baseline activation levels and/or lower quality lexical representations, cannot compete as strongly for activation; they require more time to surpass the activation and overcome the lateral inhibition of their higher-frequency counterparts. Together, these lexical candidates send negative or inhibitory feedback to the orthographic unit level, ultimately impeding the target word’s lexical accessibility. Accordingly, visual word recognition is mediated by competition and inhibition from visually similar word forms.

Although the IA model predicts inhibitory orthographic neighborhood density and frequency effects, it can, however, accommodate an opposite pattern of effects—that the activation of many orthographic neighbors, including higher-frequency ones, can boost lexical access of a target word (see, for example, Andrews, 1997; Holcomb et al., 2002). The activation of multiple visually similar lexical candidates could increase the mental lexicon’s overall excitation, ultimately facilitating the target word’s lexical accessibility due to top-down semantic-to-lexical excitatory feedback. This facilitation could benefit lower-frequency words in particular, as they are more difficult to identify due to their lower baseline activation levels and/or lower quality lexical representations. Accordingly, visual word recognition could be mediated by facilitation (rather than competition and inhibition) from visually similar word forms.

It is important to note here that the IA model was originally developed for monolingual skilled adult readers and does not make explicit predictions regarding developmental differences in orthographic neighborhood effects. On the one hand, IA may predict larger inhibitory effects in children vs. adults. Given their younger age and developing language abilities, children’s lexical representations have not benefited from as much print exposure (and language experience more generally). As a result, their words likely have differentially lower baseline activation levels and/or lower quality lexical representations, rendering them more susceptible to the effects of competition and inhibition from visually similar lexical candidates, particularly when they are higher-frequency. On the other hand, IA may also predict larger inhibitory effects in adults vs. children. Given that their lexical representations are more complex and interconnected, competition and inhibition from visually similar lexical candidates may be more pronounced due to the greater number of activated candidates.

Alternative age-related predictions are, however, possible. The activation of multiple visually similar lexical candidates, especially when lexical representations are not as entrenched in semantic memory, could accelerate the identification of orthographic units contained within target words, which, in turn, could accelerate the familiarity and overall recognition of target words. This may be particularly true for lower-frequency words, which are much less familiar to children. This would lead to larger facilitatory orthographic neighborhood density effects in children vs. adults. However, the opposite pattern may prove true: adults’ activation of a greater number of lexical candidates could lead to larger facilitatory orthographic neighborhood density effects.

#### Empirical Literature

Studies of monolingual orthographic neighborhood effects, which can be divided into those that have employed response-based tasks (including those with concurrent electroencephalographic/EEG recording) and those that have employed eye movement measures of reading, have provided mixed support for the IA model. These two categories of studies are discussed in turn, with a focus on findings involving monolingual young adults, followed by those involving monolingual children.

##### Findings From Response-Based Literature

Numerous response-based studies involving healthy monolingual young adults (aged 18–30) have reported mixed patterns of facilitatory, inhibitory, and null orthographic neighborhood density effects (e.g., Coltheart et al., 1977; Andrews, 1989, 1992; Grainger, 1992; Forster and Shen, 1996; Grainger and Jacobs, 1996; Carreiras et al., 1997; Perea and Pollatsek, 1998; Davis and Taft, 2005, Experiment 1; Pollatsek et al., 1999; Perea and Rosa, 2000, Experiment 1; Snodgrass and Mintzer, 1993; Sears et al., 1995; for reviews, see Andrews, 1997; Mathey, 2001). These between-study differences are likely driven by methodology-related factors, including the measure of orthographic neighborhood density (e.g., substitution neighbors vs. total neighbors) and the type of task used (e.g., lexical decision vs. perceptual identification tasks, which generally yield facilitatory vs. inhibitory effects, respectively, for reviews, see Andrews, 1997; Perea and Rosa, 2000; Frances et al., 2021)^1^. Moreover, only some studies have accounted for orthographic neighborhood frequency, namely, the presence of higher-frequency neighbors, which generally yields inhibitory effects (e.g., Grainger and Jacobs, 1996; Carreiras et al., 1997; Davis and Taft, 2005). In addition to methodology-related factors, some studies have also found that individual differences in reading and spelling abilities modulate orthographic neighborhood effects (e.g., Andrews and Hersch, 2010; Andrews and Lo, 2012), important factors that are rarely considered among monolingual, native language readers. Thus, support for IA is indeed very mixed.

Though relatively few in number, response-based studies involving healthy monolingual children (aged 7–12) have largely reported facilitatory orthographic neighborhood density effects (e.g., Laxon et al., 1988, 1994, 2002; Castles et al., 2003; Ziegler et al., 2003; Duñabeitia and Vidal-Abarca, 2008; but see Tamura et al., 2017 for lexical competition effects between newly learned words and their neighbors during a masked priming lexical decision task) (see text footnote 1). However, null orthographic neighborhood density effects can emerge when higher-frequency neighbors are accounted for (e.g., Marinus and de Jong, 2010).

Although direct comparisons with young adults’ orthographic neighborhood density effects have yet to be made, there is some evidence to suggest larger effects in children. For example, some studies have found that younger children make more lexicalizations than older children and young adults when presented with non-words that resemble words, such as *cholocate* vs. *chocolate* (e.g., Sebastián-Gallés and Parreño, 1995; Perea and Fraga, 2006; Perea and Estévez, 2008). Such effects can be attributed to some developing readers’ holistic strategies that rely more on coarse-grained orthographic codes (as opposed to fine-grained ones) during visual word recognition. More specifically, holistic strategies lean on minimal orthographic units needed to convey word identity, regardless of exact letter ordering (see Grainger and Ziegler, 2011)—strategies that may ultimately contribute to larger facilitatory orthographic neighborhood effects in children compared to adults. Though such a pattern of findings would refute IA’s original predictions, it can, however, be explained through the alternative interpretation of the model (discussed previously). We note, however, that children’s use of such strategies may vary as a function of the orthographic transparency of their known languages, with a potentially greater use when reading in opaque languages due to inconsistent grapheme-to-phoneme correspondences (e.g., Ziegler and Goswami, 2006; Rau et al., 2015, 2016).

##### Findings From Eye Movement Literature

Surprisingly, few monolingual studies have employed eye movement measures of reading to examine orthographic neighborhood effects, despite having several advantages over response-based tasks. These include contextualized stimuli, such as sentences and passages, instead of isolated words; naturalistic or ecologically valid tasks, such as reading for comprehension, instead of making artificial decisions to target words; and greater temporal sensitivity—being able to examine both early and late stages of word processing through different measures, such as gaze duration (i.e., the sum of all fixation durations on a word during the first pass, reflecting lexical access) and total reading time (i.e., the grand sum of all fixation durations on a word, reflecting post-lexical integration), instead of measuring global reaction times and accuracy scores for target words (Rayner, 1998, 2009). As a result, response-based tasks and eye movement measures of reading probe fundamentally different language processes (see Kuperman et al., 2013).

To date, all eye movement studies have focused on healthy monolingual young adults, and only one has examined orthographic neighborhood density effects during sentence reading (Pollatsek et al., 1999, Experiment 2). It found inhibitory effects during both early stage (gaze duration) and late-stage (total reading time) word processing. However, when higher-frequency neighbors were accounted for, the effects were facilitatory during early stage word processing (skipping rate—i.e., the probability of fixating a word during the first pass), but inhibitory during late-stage word processing (regressions out—i.e., backward eye movements to a word indicative of rereading). This suggests that the activation of multiple lexical candidates may have led participants to misread or misidentify words on the first pass, as facilitatory effects during lexical access were followed by inhibitory effects during post-lexical integration. Other eye movement studies have also reported inhibitory effects of higher-frequency neighbors (Perea and Pollatsek, 1998; Slattery, 2009; Gregg and Inhoff, 2016; but see Sears et al., 2006 for null effects). Accordingly, it appears that monolingual orthographic neighborhood effects are largely inhibitory during sentence reading—findings that support IA. However, the nature of these effects among monolingual children is currently unknown. The current study, which includes both monolingual age groups, will fill this crucial gap in the empirical literature.

### Bilingual Orthographic Neighborhood Effects

#### Theoretical Framework

Bilingualism has important consequences for how word forms are represented and retrieved from the mental lexicon during first-language (L1) and second-language (L2) visual word recognition. One of these consequences is the automatic, non-selective activation of both target and non-target language lexical representations—a phenomenon called cross-language activation. In other words, even in unilingual language contexts, bilinguals must access and retrieve correct lexical representations from a pool of visually similar lexical candidates across their known languages.

Leading theories of bilingual visual word recognition, such as the Bilingual Interactive Activation (BIA; Dijkstra and van Heuven, 1998) and Bilingual Interactive Activation Plus (BIA+; Dijkstra and van Heuven, 2002) models, which are bilingual adaptations of the monolingual IA model, propose that bilinguals have an integrated lexicon, wherein both their languages are represented. As a result, when bilinguals are visually presented with a word, similar lexical candidates from their known languages are coactivated due to spreading activation during bottom-up processing (e.g., identification of orthographic units). The activation of these lexical candidates can facilitate or inhibit word recognition, depending on a variety of factors. These include methodology-related factors, such as the nature of the task (e.g., making word judgments vs. reading for comprehension), nature of the cross-linguistic overlap (e.g., cognates, interlingual homographs, or cross-language orthographic neighbors), degree of contextual constraint (e.g., isolated words vs. sentences or paragraphs), and global language context (e.g., instructions and/or stimuli presented in the L1, L2, or both). These also include participant-related factors, such as age and manner of L1/L2 acquisition, L1/L2 dominance, L1/L2 proficiency, and domain-general executive control abilities (for reviews, see Van Assche et al., 2012; Kroll et al., 2016; Titone et al., 2016; Whitford et al., 2016; Lauro and Schwartz, 2017).

Regarding orthographic neighborhood effects, the BIA/BIA+ models make similar predictions as those of the IA model. The activation of many orthographic neighbors both within and across languages impedes lexical access of a target word. This is especially true for higher-frequency L1 orthographic neighbors, which have higher baseline activation levels and/or stronger links between different types of word-related information, such as orthography, phonology, and semantics (see Gollan et al., 2008, 2011 for a discussion of the weaker links hypothesis—a bilingual adaptation of Perfetti’s lexical quality hypothesis). Again, this impedance is attributed to lateral inhibition, which is heightened for lower-frequency L2 words; they have not benefited from as much experience, resulting in lower baseline activation levels and/or weaker links between word-related information, and, ultimately, a reduced capacity to compete with activation of their higher-frequency L1 counterparts (which send the most lateral inhibition). Although these models predict inhibitory orthographic neighborhood effects, they can, however, accommodate facilitatory ones. The activation of many orthographic neighbors (both within and across languages) could increase the mental lexicon’s overall excitation and facilitate a target word’s accessibility due to top-down semantic-to-lexical excitatory feedback, especially when it is more difficult to identify, as is the case with lower-frequency L2 words.

Like the IA model, the BIA/BIA+ models also do not make explicit predictions regarding developmental differences in orthographic neighborhood effects. However, their predictions would likely be similar. One possibility is larger inhibitory effects in children vs. adults, especially for lower-frequency L2 words. Again, this may be driven by children’s reduced age, print exposure, and language abilities, particularly in their weaker language: L2. Another possibility is larger inhibitory effects in adults vs. children, again, due to the greater competition and inhibition that ensues when more lexical candidates are activated. These factors could, however, contribute to larger facilitatory effects in these age groups, respectively. Regarding larger facilitatory effects in children, lexical representations that are not as entrenched in the mental lexicon, as is the case for lower-frequency L2 words among developing readers, could differentially benefit from the activation of multiple orthographic neighbors, thereby boosting their lexical accessibility. Regarding larger facilitatory effects in adults, the activation of a greater number of lexical candidates could boost overall lexical accessibility, which would particularly benefit the recognition of lower-frequency L2 words.

#### Empirical Literature

Studies of bilingual orthographic neighborhood effects, which can also be divided into those that have employed response-based tasks (including those with concurrent EEG recording) and those that have employed eye movement measures of reading, have provided mixed support for the BIA/BIA+ models. These two categories of studies are discussed in turn, with a focus on findings involving bilingual young adults. Although the current study represents the first investigation of orthographic neighborhood effects in bilingual children, relevant findings from studies investigating other aspects of orthographic processing are discussed.

##### Findings From Response-Based Literature

The bilingual literature parallels the monolingual literature; it has reported mixed patterns of facilitatory, inhibitory, and null within-language and cross-language orthographic neighborhood density effects among healthy bilingual young adults (aged 18–30) across their L1 and L2 (e.g., Beauvillain, 1992; de Groot et al., 2002; Dirix et al., 2017, Commissaire et al., 2019, Experiment 1; Grainger and Dijkstra, 1992; Van Heuven et al., 1998; Lemhöfer et al., 2008; Midgley et al., 2008; Grossi et al., 2012; Meade et al., 2018; Mulder et al., 2018). As discussed previously, these between-study differences are likely driven by methodology-related factors, including whether orthographic neighborhood frequency was accounted for, as well as by participant-related factors. Thus, support for BIA/BIA+ is indeed very mixed.

Although no prior response-based studies have investigated orthographic neighborhood effects in bilingual children, there is evidence that their visual word recognition is differentially mediated by cross-language orthographic overlap, especially when they are younger in age (for a review, see van Hell, 2020). For instance, Duñabeitia et al. (2016) tested a large sample (*N* = 100) of balanced Spanish-Basque bilingual children (aged 8–15) on L1 and L2 translation recognition tasks and found that the younger children’s performance was more sensitive to the target words’ cognate status, with greater orthographic overlap facilitating word recognition (see also Schröter and Schroeder, 2016, 2018 and Duñabeitia et al., 2020, for similar findings involving similar and other aspects of cross-language orthographic processing in children during L1 and L2 lexical decision tasks). These effects have been attributed to younger children’s reduced print exposure and developing language control systems, which render their lexical representations more susceptible to the effects of cross-language activation. Based on this work, larger facilitatory orthographic neighborhood effects in bilingual children vs. adults are likely. Such a pattern of findings would support the alternative interpretation of BIA/BIA+ (discussed previously).

##### Findings From Eye Movement Literature

Though less than a handful, eye movement studies of reading have generally reported facilitatory within-language and cross-language orthographic neighborhood density effects among healthy bilingual young adults (aged 18–30) across their L1 and L2. Thus, these findings refute BIA/BIA+’s original predictions and suggest that the models may require modifications to account for certain aspects of within-language and cross-language activation during natural reading. Indeed, these findings support the models’ alternative interpretation.

In the earliest of studies, Whitford et al. (2016) found facilitatory cross-language orthographic neighborhood density effects during L1 and L2 paragraph reading in a large sample (*N* = 117) of balanced English-French bilingual young adults. Words with many vs. fewer cross-language neighbors were easier to process, evidenced by shorter gaze durations and total reading times. This was especially true for lower-frequency L2 words, which have differentially lower baseline activation levels and/or weaker links between word-related information. The patterns of within-language orthographic neighborhood density effects differed across the L1 and L2; they were null vs. facilitatory. The study did, however, have two important limitations that were addressed in subsequent work: it only included substitution neighbors and did not account for orthographic neighborhood frequency.

Consistent with Whitford et al.’ (2016) study, Dirix et al. (2017, Experiment 2) also found largely facilitatory cross-language orthographic neighborhood density effects during L1 and L2 novel reading in a small sample (*N* = 19) of unbalanced Dutch-English bilingual young adults. Although the effects were rather limited during L1 reading, words with many vs. fewer cross-language orthographic neighbors were, again, processed more easily, evidenced by shorter gaze durations and total reading times. The patterns of within-language neighborhood density effects differed across the L1 and L2. In the L1, they were facilitatory for lower-frequency words and inhibitory for higher-frequency words, whereas in the L2, they were entirely facilitatory.

Extending their previous study to examine age differences in orthographic neighborhood density effects, Whitford and Titone (2019) found facilitatory within-language and cross-language neighborhood density effects during L1 and L2 paragraph reading in large samples (*n* = 62 each) of balanced French-English bilingual younger and older adults (aged 60+), matched on gender, education, L1/L2 background, and L1/L2 proficiency (both objective and subjective). Although their findings patterned with those of their earlier study, larger effects were observed among older adults. Thus, despite having benefited from more life-long print exposure (and language experience more generally), older adults’ lexical accessibility may be negatively mediated by age-related changes in cognitive and sensory processing.

Although no prior eye movement reading studies have investigated orthographic neighborhood effects in bilingual children, a recent study suggests that their visual word recognition may be positively mediated by cross-language orthographic overlap. Bosma and Nota (2020) found that a group (*N* = 37) of L2-dominant Frisian-Dutch bilingual children (aged 9–12) were sensitive to target words’ cognate status, with greater orthographic overlap (form-identical, followed by form-non-identical cognates) facilitating word recognition, evidenced by shorter gaze durations and total reading times.

Taken together, the above-reviewed bodies of literature suggest that visual word recognition is influenced by orthographic neighborhood effects in various ways across different experimental tasks and participant groups. Here, we clarify and unify these distinct bodies of literature by examining how both within-language and cross-language orthographic neighborhood effects influence visual word recognition during naturalistic reading in different age groups (children, adults) and language groups (monolinguals, bilinguals), and whether the observed findings can be captured by the IA and BIA/BIA+ models. Thus, this work will further our understanding of a relatively understudied potential moderator of within-language and cross-language activation in diverse groups of people: orthographic neighborhood density.

### The Current Study

We investigated how monolingual and bilingual children’s and young adults’ L1 and L2 eye movement reading behavior was influenced by orthographic neighborhood density (both cross-language and within-language, where applicable), while controlling for the presence of higher-frequency orthographic neighbors. Based on previous findings from the bilingual eye movement reading literature (which are largely consistent with BIA/BIA+’s alternative explanations), we predicted facilitatory cross-language and within-language orthographic neighborhood density effects across both early and late reading stages: words with higher orthographic neighborhood densities should be easier to process, evidenced by shorter gaze durations and total reading times. However, we also predicted modulatory effects of age group and language group based on the lower word baseline activation levels and/or weaker links that some of the participant groups may experience (namely, bilinguals and children). Thus, our specific hypotheses were as follows:

(1) During L1 reading, larger facilitatory within-language (L1) effects in bilinguals vs. monolinguals, as well as in children vs. adults.
(2) During L2 reading, larger facilitatory within-language (L2) effects in bilingual children vs. bilingual adults.
(3) During L1 reading, larger cross-language (L2) effects in bilingual children vs. bilingual adults.
(4) During L2 reading, larger cross-language (L1) effects in bilingual children vs. bilingual adults.

## Materials and Methods

### Participants

Participants were the same as those included in Whitford and Joanisse (2018). They comprised four groups: (1) English monolingual children aged 7–12 (*n* = 34); (2) English-French bilingual children aged 7–12 (*n* = 33); (3) English monolingual adults aged 18–21 (*n* = 30); and (4) English-French bilingual adults aged 18–21 (*n* = 30). The children were recruited from English-language, French-language, and French immersion elementary schools in London, Ontario, Canada, and the adults were recruited from Western University (most of the bilingual adults attended French immersion schools as children). All participants had English as their first acquired and dominant language (L1), and all bilingual participants had French as their second acquired and weaker language (L2). Note that some of the monolingual children and adults had some French instruction through the Ontario educational curriculum; however, all self-identified as functionally monolingual. All participants were typically developing, with no uncorrected visual or hearing impairments, and no language, learning, neurological, or psychiatric disorders. The study was part of a larger experimental protocol that lasted 3 hours. Participants received a $30 movie gift card or course credit as compensation. The study was approved by Western University’s Non-Medical Research Ethics Board (106319/106601).

Participants completed three background measures. First, adaptations of the Language Experience and Proficiency Questionnaire (LEAP-Q; Marian et al., 2007) were used to assess participants’ demographic and language backgrounds, including L1/L2 age of acquisition (AoA) and current L1/L2 exposure. Second, the Word Reading and Pseudoword Decoding subtests of the Wechsler Individual Achievement Test—Second Edition (WIAT-II; English-Canadian and French-Canadian adaptations; Wechsler, 2005) were used to assess participants’ L1 and L2 word-level reading skills. More specifically, the Word Reading subtest measured accuracy of word recognition without contextual clues and the Pseudoword Decoding subtest measured accuracy of deciphering non-sense words. Participants read aloud a list of words (maximum: 131) and a list of made-up words (maximum: 55) that increased in difficulty. Raw subtest scores were converted to age-based standard scores (*M* = 100 ± 15). Third, the Test of Non-Verbal Intelligence—Third Edition (TONI-III; Brown et al., 1997) was used to assess participants’ non-verbal IQ. More specifically, participants completed sequences of shapes (maximum: 45) by selecting one option among six possible response options. Raw scores were converted to age-based standard scores (*M* = 100 ± 15).

Participant characteristics are presented in Tables 1, 2, which demonstrate that the two groups of children and the two groups of adults were matched on age, sex, education, parental socioeconomic status (SES) based on the Hollingshead Occupational Scale (Hollingshead, 1975), non-verbal IQ, self-report (i.e., LEAP-Q) measures of L1 history and proficiency, and objective (i.e., WIAT-II) measures of L1 reading ability (all *p-*values > 0.5). Expectedly, both monolingual groups had significantly lower L2 proficiency than their bilingual counterparts based on self-report measures (all *p*-values < 0.001); the monolingual groups (especially the children) lacked the proficiency needed to complete the objective measures. Both bilingual groups had significantly lower L2 vs. L1 proficiency based on both self-report and objective measures (all *p-*values < 0.05). Although the two groups of children were matched as closely as possible to their adult counterparts, including on parental SES and WIAT-II Pseudoword Decoding (across their known languages), they significantly differed on a number of other measures, including non-verbal IQ, L1 WIAT-II Word Reading, and current L1/L2 exposure (for the bilingual groups) (all *p-*values < 0.01). Thus, we decided to control for these differences in our analyses. Nonetheless, the pattern of results (reported subsequently) remained unchanged even when subsets of adults matched even more closely to the groups of children were included in the analyses.

**TABLE 1:** Characteristics of the child participant groups.

	**Monolingual children (*n* = 34) [mean (*SD*)]**	**Bilingual children (*n* = 33) [mean (*SD*)]**
Age (years)	9.82 (1.10)	10.02 (1.32)
Sex (male:female ratio)	14:20	13:20
Education (years)	4.09 (1.08)	4.21 (1.39)
Parental SES^a^	3.00 (1.18)	2.88 (1.36)
TONI-III (standard scores)	109.88 (17.10)	117.18 (18.04)
**AoA; Age of fluency (years)**		
L1	Birth (−); 2.71 (0.95)	Birth (−); 2.43 (1.17)
L2***	7.42 (1.82); Never (−)	3.82 (1.66); 5.57 (1.96)
**Reading AoA; Age of reading fluency (years)**		
L1	4.35 (0.96); 6.05 (0.95)	4.48 (1.14); 6.23 (1.37)
L2***	8.28 (1.07); Never (−)	5.47 (1.05); 7.36 (1.44
**Current language exposure (% time)**		
L1***	95.53 (5.66)	58.03 (12.93)
L2***	4.47 (5.66)	39.70 (13.11)
**Current reading exposure (% time)**		
L1***	99.79 (0.88)	65.30 (25.98)
L2***	0.21 (0.88)	33.58 (25.35)
**L1 self-report proficiency measures (1–7)^b^**		
Reading ability	6.06 (1.41)	5.64 (1.41)
Overall competence	6.15 (1.31)	5.88 (1.11)
**L2 self-report proficiency measures (1–7)^b^**		
Reading ability***	1.06 (0.24)	4.58 (1.28)
Overall competence***	1.06 (0.24)	4.67 (1.31)
**L1 WIAT-II (standard scores)**		
Word Reading	99.44 (12.58)	99.15 (17.38)
Pseudoword Decoding	106.26 (15.61)	103.12 (17.22)
**L2 WIAT-II (standard scores)**		
Word Reading	−	88.55 (23.77)
Pseudoword Decoding	−	95.70 (20.73)

*SES, socioeconomic status; TONI-III, Test of Non-Verbal Intelligence—3rd Edition; AoA, age of acquisition; L1, first-language; L2, second-language; WIAT-II, Wechsler Individual Achievement Test—2nd Edition.*

*^*a*^Scale from 1 (major professional) to 9 (unemployed).*

*^*b*^Scale from 1 (beginner) to 7 (native-like).*

***p < 0.001.

**TABLE 2:** Characteristics of the adult participant groups.

	**Monolingual adults (*n* = 30) [mean (*SD*)]**	**Bilingual adults (*n* = 30) [mean (*SD*)]**
Age (years)	18.67 (0.94)	18.33 (0.60)
Sex (male:female ratio)	10:20	5:25
Education (years)	13.35 (0.52)	13.17 (0.30)
Parental SES^a^	2.63 (1.14)	2.27 (1.09)
TONI-III (standard scores)	99.60 (11.84)	99.39 (14.04)
**AoA; Age of fluency (years)**		
L1	Birth (−); 3.72 (1.80)	Birth (−); 3.68 (1.75)
L2***	8.96 (2.46); Never (−)	5.53 (2.42); 10.95 (4.51)
**Reading AoA; Age of reading fluency (years)**		
L1	4.52 (1.32); 6.50 (1.96)	5.03 (1.49); 7.07 (1.57)
L2***	9.90 (2.24); Never (−)	7.13 (2.08); 11.10 (3.56)
**Current language exposure (% time)**		
L1**	99.70 (0.97)	86.41 (19.71)
L2***	0.30 (0.97)	12.73 (19.67)
**Current reading exposure (% time)**		
L1***	100.00 (0.00)	86.34 (18.84)
L2***	0.00 (0.00)	13.66 (18.84)
**L1 self-report proficiency measures (1–7)^b^**		
Reading ability	6.83 (0.73)	6.67 (0.74)
Overall competence	6.93 (0.36)	6.70 (0.53)
**L2 self-report proficiency measures (1–7)^b^**		
Reading ability***	1.43 (0.62)	5.20 (0.98)
Overall competence***	1.17 (0.37)	4.83 (1.07)
**L1 WIAT-II (standard scores)**		
Word Reading	111.80 (6.55)	112.43 (5.39)
Pseudoword Decoding	105.73 (11.85)	109.07 (8.12)
**L2 WIAT-II (standard scores)**		
Word Reading	−	81.18 (18.92)
Pseudoword Decoding	−	97.70 (12.78)

*SES, socioeconomic status; TONI-III, Test of Non-Verbal Intelligence—3rd Edition; AoA, age of acquisition; L1, first-language; L2, second-language; WIAT-II, Wechsler Individual Achievement Test—2nd Edition.*

*^*a*^Scale from 1 (major professional) to 9 (unemployed).*

*^*b*^Scale from 1 (beginner) to 7 (native-like).*

**p < 0.01; ***p < 0.001.

### Materials

Stimuli were the same as those in Whitford and Joanisse (2018): English and French versions of four paragraphs (two fiction and two non-fiction short stories), drawn from the Reading Comprehension subtest of the WIAT-II (English-Canadian and French-Canadian adaptations; Wechsler, 2005). The paragraphs were representative of those read in elementary educational settings across Canada; thus, they had a high degree of ecological validity. The English and French versions of the paragraphs contained a comparable number of words (105, 87, 103, and 195 words vs. 118, 95, 109, and 200 words). Important lexical characteristics were obtained for the words of each paragraph, including length, frequency, predictability, mean bigram frequency (both within-language and cross-language), total phonological neighborhood density (both within-language and cross-language), total orthographic neighborhood density (both within-language and cross-language), orthographic neighborhood density of higher-frequency neighbors (both within-language and cross-language), and orthographic neighborhood frequency of higher-frequency neighbors (both within-language and cross-language).

The paragraphs’ English and French lexical characteristics were obtained as follows. Subtitle word frequency values (in occurrences per million words) were gathered from SUBTLEX-US (Brysbaert and New, 2009) via the English Lexicon Project (Balota et al., 2007) and Lexique (New et al., 2001), respectively. Mean bigram frequencies were computed by dividing summated bigram frequencies from WordGen (Duyck et al., 2004) by word length (following Dirix et al., 2017). Word predictability values were derived through computerized cumulative cloze tasks involving separate samples of native English (*n* = 30) and native French (*n* = 30) young adult participants, who guessed the words of each paragraph one at a time, until the entire paragraph was presented on the computer screen (following Miellet et al., 2007; Whitford and Titone, 2012, 2014, 2017, 2019). Neighborhood density and neighborhood frequency values were gathered from the Cross-Linguistic Easy-Access Resource for Phonological and Orthographic Neighborhood Densities (CLEARPOND; Marian et al., 2012). Paragraph characteristics are presented in Supplementary Table A1.

A total of 210 language-unique target words were selected from the paragraphs and included in the analyses. Exclusions were as follows: line-initial, line-final, function, proper noun, punctuated, and repeated words, as well as cognates and interlingual homographs (Pollatsek et al., 2006; Miellet et al., 2007; Whitford and Titone, 2012, 2014, 2017, 2019). Target word characteristics are presented in Supplementary Table A2.

### Apparatus

Right eye movements were sampled at 1 kHz using an EyeLink 1000 desktop-mounted eye-tracker (SR-Research, Ontario, Canada). The paragraphs were viewed binocularly on a 21” ViewSonic CRT monitor (screen resolution: 1,024 × 768 pixels; viewing distance: 60 cm). Depending on their length, the paragraphs were presented on one or two black display pages in yellow 14-point Courier New font using Experiment Builder (SR-Research, Ontario, Canada). Each display page had a maximum of 10 lines of text, 70 characters per line, and 2 characters per 1° of visual angle. Eye movements were calibrated with a nine-point grid (average fixation error: < 0.5° of visual angle following validation). A padded head-rest minimized head movements during reading.

### Procedure

The procedure was the same as that of Whitford and Joanisse (2018). After providing both oral and written assent and/or consent, participants read the four paragraphs (two in their L1 and two in their L2) silently and naturally for comprehension while their eye movements were monitored. Paragraph version (1, 2, 3, 4) and paragraph language (L1, L2) were counterbalanced across participants. Calibration procedures were performed before each paragraph was read. Comprehension was assessed via four open-ended, orally administered questions after reading each paragraph (total score: 16). Correct, partially correct, and incorrect answers were scored as 1, 0.5, and 0, respectively (Radach et al., 2008; Whitford and Titone, 2012, 2014, 2017, 2019). Subsequently, participants (or the caregivers/parents of children) completed the LEAP-Q, followed by the WIAT-II Word Reading and Pseudoword Decoding subtests (counterbalanced across participants) in English (if monolingual) or in both English and French (if bilingual), and the TONI-III.

## Results

### Reading Comprehension Performance

A one-way ANOVA revealed comparable L1 (English) reading comprehension accuracy between the four participant groups [*F*_(3, 123)_ = 0.47, *p* = 0.703]. Moreover, a two-way ANOVA revealed comparable L1 (English) and L2 (French) reading comprehension accuracy between the two bilingual groups [*F*_(1, 122)_ = 0.05, *p* = 0.822]. Thus, there were no between-group or between-language differences in reading comprehension performance. Means and standard deviations are presented in Table 3.

**TABLE 3:** Paragraph reading comprehension performance (% correct).

	**Monolingual children (*n* = 34) [mean (*SD*)]**	**Bilingual children (*n* = 33) [mean (*SD*)]**	**Monolingual adults (*n* = 30) [mean (*SD*)]**	**Bilingual adults (*n* = 30) [mean (*SD*)]**
L1	84.01 (11.74)	82.46 (17.54)	86.25 (11.42)	87.04 (13.10)
L2	−	77.83 (18.66)	−	81.47 (14.77)

*L1, first-language; L2, second-language.*

### Eye Movement Reading Performance

The EyeLink 1000 software identified fixations (pauses) and saccades (eye movements), which had a minimum velocity of 30°/s, minimum acceleration of 8,000°/s^2^, and minimum change in eye position of 0.15°. A lower cut-off of 80 ms was applied to all fixations (<5% of data); however, an upper cut-off was not applied to maximize data inclusion (maximum fixation duration: 2,605 ms made by a bilingual child reading in their L2).

We examined two eye movement measures. One reflected early stage reading (i.e., lexical access): gaze duration (i.e., the sum of all fixation durations on a word during the first pass). One reflected late-stage reading (i.e., post-lexical integration): total reading time (i.e., the grand sum of all fixation durations on a word). Only fixations on the 210 language-unique target words were included in the analyses.

We used linear mixed-effects models (LMMs) to analyze the data via the lme4 package (Bates, 2007; Bates et al., 2015) in version 4.0.4 of R (Baayen, 2008; R Development Core Team, 2021). We ran four models across the two eye movement measures. They examined between-group differences in: (1) within-language (L1) neighborhood density effects during L1 reading; (2) within-language (L2) neighborhood density effects during L2 reading; (3) cross-language (L2) neighborhood density effects during L1 reading; and (4) cross-language (L1) neighborhood density effects during L2 reading. The fixed effects (i.e., factors of theoretical interest), control predictors (i.e., covariates), and random effects (i.e., random intercepts and/or slopes for participants and items) for each model are reported subsequently. Across all models, categorical variables were deviation coded (−0.5, 0.5), where the mean of each level was compared to the grand mean, and continuous variables were scaled (i.e., standardized, z-scored) to reduce collinearity. Of note, only significant effects (i.e., those with |*t|* values > 1.96, corresponding to α = 0.05) involving the fixed effects and their interactions are reported subsequently; however, complete model outputs can be found in Supplementary Appendix.

#### Model 1: Within-Language (L1) Neighborhood Density Effects on L1 Reading

All four participant groups were included in this analysis. The fixed factors were age group (children vs. adults), language group (monolingual vs. bilingual), and total within-language (L1) orthographic neighborhood density (continuous). The word-related control predictors were length (continuous), frequency (continuous, log-transformed), predictability (continuous), mean within-language bigram frequency (continuous, log-transformed), total within-language phonological neighborhood density (continuous), within-language orthographic neighborhood density of higher-frequency neighbors (continuous), and within-language orthographic neighborhood frequency of higher-frequency neighbors (continuous). The participant-related control predictors were L1 WIAT-II Word Reading standard scores (continuous), current L1 exposure (continuous), and TONI-III standard scores (continuous). The random effects were random intercepts for participants and paragraph version (following Whitford and Titone, 2012, 2014, 2017, 2019). Complete model outputs for this analysis can be found in Supplementary Table A3.

The effect of age group was significant for gaze duration (β = 62.69, *SE* = 18.08, *t* = 3.47, *p* = 0.001). Children had longer gaze durations (343 vs. 251 ms) than adults, reflecting more effortful reading. The effect of total (L1) orthographic neighborhood density was near-significant for total reading time (β = −15.64, *SE* = 8.22, *t* = −1.90, *p* = 0.057). Words with many vs. few within-language neighbors were easier to process, evidenced by shorter total reading times (378 vs. 431 ms).^2^

**FIGURE 1:**
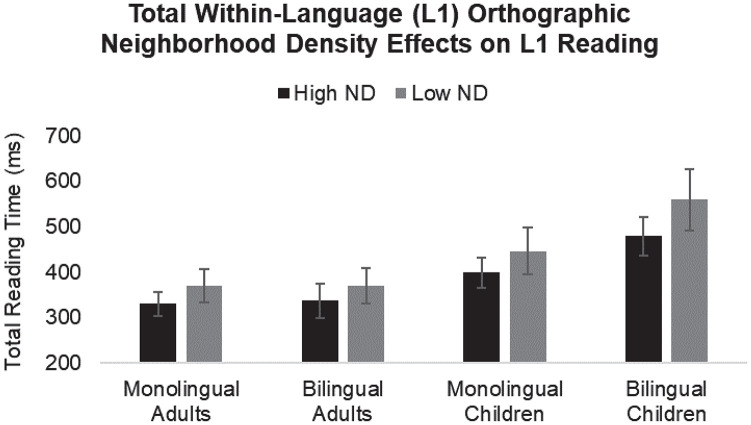
The effect of total within-language (L1) orthographic neighborhood density on the monolingual and bilingual age groups’ L1 total reading times. Means are plotted. Error bars represent ± 1 standard error of the mean.

Moreover, the three-way interaction between age group, language group, and total within-language (L1) orthographic neighborhood density was significant for total reading time (β = 41.36, *SE* = 19.46, *t* = 2.13, *p* = 0.034). To facilitate interpretation of the higher-order interaction, we ran separate follow-up models with either monolingual adults, bilingual adults, or monolingual children as the baseline. Significantly or marginally larger facilitatory neighborhood density effects were found between the following groups: monolingual children vs. monolingual adults (β = −16.86, *SE* = 8.65, *t* = −1.95, *p* = 0.052); bilingual children vs. monolingual adults (β = −38.88, *SE* = 13.60, *t* = −2.86, *p* = 0.004); monolingual children vs. bilingual adults (β = −34.08, *SE* = 13.92, *t* = −2.45, *p* = 0.014); bilingual children vs. bilingual adults (β = −57.16, *SE* = 15.97, *t* = −3.58, *p* < 0.001); and bilingual children vs. monolingual children (β = −23.08, *SE* = 13.25, *t* = −1.74, *p* = 0.082). No significant difference was found between the adult groups (β = −18.27, *SE* = 14.26, *t* = −1.28, *p* = 0.200). Thus, as can be seen in Figure 1, both groups of children exhibited larger facilitatory neighborhood density effects than both groups of adults; words were easier to process when they had many vs. few within-language neighbors, evidenced by shorter total reading times (see text footnote 2). However, the magnitude of these effects was most pronounced in bilingual children; they found words with few within-language neighbors especially difficult to process, evidenced by differentially longer total reading times.

##### Summary of Model 1’s Findings

We observed larger facilitatory total within-language (L1) orthographic neighborhood density effects in children vs. adults during late-stage reading, especially among bilingual children.

#### Model 2: Within-Language (L2) Neighborhood Density Effects on L2 Reading

Only the bilingual groups were included in this analysis. The fixed factors were age group (children vs. adults) and total within-language (L2) orthographic neighborhood density (continuous). The word-related control predictors were the same as in the previous model. The participant-related control predictors were L2 WIAT-II Word Reading standard scores (continuous), current L2 exposure (continuous), and TONI-III standard scores (continuous). The random effects were the same as in the previous model. Complete model outputs for this analysis can be found in Supplementary Table A4.

Although no effects reached significance, the effect of total within-language (L2) orthographic neighborhood density was near-significant for total reading time (β = −32.95, *SE* = 17.30, *t* = −1.90, *p* = 0.057). Words with many vs. few within-language neighbors were easier to process, evidenced by numerically shorter total reading times (491 vs. 679 ms).^2^

##### Summary of Model 2’s Findings

We observed numerically facilitatory total within-language (L2) orthographic neighborhood density effects during late-stage reading that were age-invariant.

#### Model 3: Cross-Language (L2) Neighborhood Density Effects on L1 Reading

All four participant groups were included in this analysis. The fixed factors were the same as those in Model 1, except that total cross-language (L2) orthographic neighborhood density (continuous) was included instead. The word-related and participant-related control predictors were also the same as those in Model 1, with the addition of mean cross-language bigram frequency (continuous, log-transformed), total cross-language phonological neighborhood density (continuous), cross-language orthographic neighborhood density of higher-frequency neighbors (continuous), and cross-language orthographic neighborhood frequency of higher-frequency neighbors (continuous). The random effects were the same as those in previous models. Complete model outputs for this analysis can be found in Supplementary Table A5.

The effect of age group was significant for gaze duration (β = 45.67, *SE* = 21.72, *t* = 2.10, *p* = 0.037). Children had longer gaze durations than adults (343 vs. 251 ms), reflecting more effortful reading. Moreover, the effect of total cross-language (L2) orthographic neighborhood density was significant for both gaze duration (β = −31.43, *SE* = 10.86, *t* = −2.90, *p* = 0.006) and total reading time (β = −43.88, *SE* = 16.64, *t* = −2.64, *p* = 0.008). Words with many vs. few cross-language neighbors were easier to process, evidenced by shorter gaze durations (276 vs. 306 ms) and total reading times (370 vs. 430 ms) (see text footnote 2). The interactions with age group and language group were non-significant for both eye movement measures.

##### Summary of Model 3’s Findings

We observed facilitatory total cross-language (L2) orthographic neighborhood density effects across both reading stages that were age-invariant and language background-invariant.

#### Model 4: Cross-Language (L1) Neighborhood Density Effects on L2 Reading

Only the bilingual groups were included in this analysis. The fixed factors were the same as those in Model 2, except that total cross-language (L1) orthographic neighborhood density (continuous) was included instead. The word-related and participant-related control predictors were also the same as those in Model 2, with the addition of mean cross-language bigram frequency (continuous, log-transformed), total cross-language phonological neighborhood density (continuous), cross-language orthographic neighborhood density of higher-frequency neighbors (continuous), and cross-language orthographic neighborhood frequency of higher-frequency neighbors (continuous). The random effects were the same as those in previous models. Complete model outputs for this analysis can be found in Supplementary Table A6.

The effect of total cross-language (L1) orthographic neighborhood density significant for gaze duration (β = −144.19, *SE* = 65.34, *t* = −2.21, *p* = 0.032). Words with many vs. few within-language neighbors were easier to process, evidenced by shorter gaze durations (290 vs. 425 ms) (see text footnote 2). The interaction with age group was non-significant for both eye movement measures.

##### Summary of Model 4’s Findings

We observed facilitatory total cross-language (L1) orthographic neighborhood density effects during early stage reading that were age-invariant.

## Discussion

While engaging in visual word recognition, individuals must access and retrieve correct lexical representations from their mental lexicon among an array of visually similar lexical candidates: orthographic neighbors. While this process is limited to the activation of within-language orthographic neighbors among monolinguals, it is more complex among bilinguals due to the simultaneous activation of both within-language and cross-language orthographic neighbors. Thus, bilingual visual word recognition is influenced by the non-selective activation of both target and non-target language lexical representations, even in unilingual contexts.

While much is known about orthographic neighborhood effects among monolingual young adults, far less is known among other age and language groups (e.g., children, bilinguals). With the overarching aim of developing a more comprehensive understanding of within-language and cross-language activation during naturalistic reading in diverse groups of people, the current study employed eye movement measures to examine how within-language and cross-language orthographic neighborhood density influence visual word recognition during L1 and L2 paragraph reading in groups of monolingual and bilingual children and young adults. We had four main findings: two pertained to L1 and L2 within-language effects and the other two pertained to L1 and L2 cross-language effects. Each finding is discussed in turn.

### Within-Language (L1) Neighborhood Density Effects on L1 Reading

Our first main finding was that high within-language (L1) orthographic neighborhood densities facilitated late-stage L1 word processing across all participant groups. Words with many within-language orthographic neighbors received shorter total reading times than those with fewer neighbors. However, the magnitude of these effects was larger in children, particularly the bilingual ones. Consistent with our original hypothesis, this finding suggests that the activation of multiple visually similar word forms facilitates target word recognition, particularly under conditions of low lexical entrenchment (i.e., when words have lower baseline activation levels and/or weaker links between different types of word-related information, as is likely the case for children and bilinguals). Given their reduced age, children’s lexical representations have not benefited from as much life-long language exposure as those of young adults (their language and cognitive skills are still developing). Similarly, given their divided L1/L2 exposure, bilinguals’ lexical representations have not benefited from as much absolute exposure as those of monolinguals. As a result, both conditions may entail reduced lexical entrenchment, evidenced by reduced ease of word processing. Combined, these conditions may engender a “double whammy,” as evidenced by bilingual children’s differentially reduced ease of word processing (see Figure 1). Accordingly, readers may maximally capitalize on high orthographic neighborhood densities under such conditions to identify the orthographic patterns of target words and retrieve their meaning from the mental lexicon, in an effort to offset their reduced lexical accessibility (see, for example, Laxon et al., 1988, 1994, 2002; Duñabeitia and Vidal-Abarca, 2008; for response-based studies reporting similar patterns among monolingual children). Indeed, there were no between-group differences in our participants’ reading comprehension performance (the ultimate goal of reading), suggesting a compensatory reading strategy (see also Whitford and Titone, 2019, for a similar strategy among bilingual older adults during paragraph reading).

Although these findings do not support IA and BIA/BIA+’s original predictions (i.e., inhibitory orthographic neighborhood density effects, particularly for words with lower baseline activation levels and/or weaker links due to lateral inhibition from words with higher baseline activation levels and/or stronger links), they support their alternative interpretation: that the activation of multiple lexical candidates can boost the overall excitation of the mental lexicon which, in turn, can boost the familiarity and activation levels of target words.

With regard to the extant eye movement literature, our findings are inconsistent with the few monolingual studies that have reported inhibitory orthographic neighborhood effects during sentence reading in adults (e.g., Pollatsek et al., 1999, Experiment 2; Slattery, 2009). Rather, they are consistent with the few bilingual studies that have reported largely facilitatory within-language orthographic neighborhood effects during L1 novel (Dirix et al., 2017, Experiment 2) and paragraph (Whitford et al., 2016; Whitford and Titone, 2017, 2019) reading in adults. Thus, similar to the monolingual and bilingual response-based literatures, which have reported mixed patterns of facilitatory, inhibitory, and null orthographic neighborhood effects, as a function of different word processing tasks, our findings suggest that such effects can also differ during naturalistic reading, as a function of different reading tasks and goals. For example, reading numerous short unrelated sentences, followed by simple yes/no comprehension questions on a percentage of trials, could contribute to inhibitory effects, whereas reading lengthy paragraphs of text that place greater demands on the visual, executive functioning, and linguistic systems, followed open-ended comprehension questions, could contribute to facilitatory effects. As such, readers may capitalize more on high orthographic neighborhood densities during more effortful reading conditions to offset the greater processing demands, a strategy similar in principle to that proposed earlier for the bilingual children.

### Within-Language (L2) Neighborhood Density Effects on L2 Reading

Our second main finding was that within-language (L2) neighborhood density effects were equivocal. Although we observed numerically facilitatory effects during late-stage L2 word processing across both age groups—a pattern that would support an alternative interpretation of BIA/BIA+ that can accommodate facilitatory within-language orthographic neighborhood effects, as well as the extant bilingual eye movement literature (Dirix et al., 2017, Experiment 2; Whitford et al., 2016; Whitford and Titone, 2017, 2019)—if real, the effects are likely weak and may require more tightly controlled stimuli to isolate them. Thus, future work in this area is needed.

### Cross-Language (L2) Neighborhood Density Effects on L1 Reading

Our third main finding was that high cross-language (L2) orthographic neighborhood densities facilitated both early stage and late-stage L1 word processing across all participant groups. Words with many cross-language orthographic neighbors received shorter gaze durations and total reading times than those with fewer neighbors. Although we predicted larger facilitatory effects in bilingual children vs. bilingual adults, no interactions with age group or language group reached significance. We would like to highlight here that despite self-identifying as functionally monolingual, most of our monolingual participants did, however, have some minimal L2 (French) proficiency; they completed basic French courses through the Ontario educational curriculum. Thus, it is possible that their L2 proficiency was sufficient enough to experience cross-language activation of visually similar L2 word forms. We note, however, that the magnitude of this cross-language activation was numerically smaller than that experienced by the bilingual participants. A closer look at the participant group means (based on a median splits) (see text footnote 2), revealed larger facilitative effects among bilingual children (56 ms), followed by bilingual adults (28 ms), monolingual children (22 ms), and monolingual adults (16 ms). Nonetheless, we cannot rule out the possibility that these effects were driven by some other, uncontrolled factor.

On the whole, these findings support an alternative interpretation of BIA/BIA+ that can accommodate facilitatory cross-language orthographic neighborhood effects and are largely consistent with previous eye movement studies of orthographic neighborhood effects in bilingual adults (Dirix et al., 2017, Experiment 2; Whitford et al., 2016; Whitford and Titone, 2017, 2019).

### Cross-Language (L1) Neighborhood Density Effects on L2 Reading

Our fourth main finding was that high cross-language (L1) orthographic neighborhood densities facilitated early stage L2 word processing across both bilingual age groups. Again, words with many cross-language orthographic neighbors received shorter gaze durations than those with fewer neighbors. However, late-stage L2 word processing (i.e., total reading time) was not affected, suggesting that facilitation (potentially due to top-down semantic-to-lexical excitatory feedback) occurred sufficiently so during first pass reading of the target words. Although we also predicted larger facilitatory effects in children vs. adults, the magnitude was age-invariant. This is likely because the two bilingual age groups were matched (all *p*-values > 0.05) on objective measures of L2 reading proficiency: L2 WIAT-II Word Reading standard scores (88.55 vs. 81.18) and L2 WIAT-II Pseudoword Decoding standard scores (95.70 vs. 97.70). Moreover, despite having accrued less life-long L2 exposure, the children had significantly higher current L2 exposure levels than the adults (39.70 vs. 12.73%; *p* < 0.001). Thus, greater “in the moment” L2 experience levels or more “bilingual modes” could counteract any historically driven age-related differences in visual word recognition.

As with the other orthographic neighborhood density effects discussed earlier, these findings also support an alternative interpretation of BIA/BIA+ and are consistent with previous eye movement studies of orthographic neighborhood effects in bilingual adults (Dirix et al., 2017, Experiment 2; Whitford et al., 2016; Whitford and Titone, 2017, 2019).

## Conclusion

The current study represents the first systematic investigation of within-language and cross-language activation during reading by means of orthographic neighborhood effects in a number of relatively understudied groups, including monolingual children, bilingual children, and bilingual adults. This work makes important empirical and theoretical contributions to the field by demonstrating that visually similar word forms, both within and across languages, facilitates visual word recognition during reading conditions that resemble those encountered in everyday life. Future avenues of research should explore whether leading models of visual word recognition, such as IA and BIA/BIA+, which were originally developed for skilled adult readers processing isolated words, can simulate the observed pattern of findings.

## Data Availability Statement

The data analyzed in this study is subject to the following licenses/restrictions: The stimuli used in the study were copyrighted material: passages from the Reading Comprehension subtest of the Weschler Individual Achievement Test—Second Edition (WIAT-II; Weschler 2005). However, the data will be made available upon request by contacting the corresponding author.

## Ethics Statement

The study involved human participants and was approved by Western University’s Research Ethics Board (REB). Written informed consent to participate in this study was provided by the participants’ legal guardian/next of kin.

## Author Contributions

VW designed the experiment, collected, processed, and analyzed the data, and wrote the manuscript. MFJ provided direct funding for the research and feedback during various stages of the research process, including critically evaluating the manuscript. Both authors contributed to the article and approved the submitted version.

## Conflict of Interest

The authors declare that the research was conducted in the absence of any commercial or financial relationships that could be construed as a potential conflict of interest.

## Publisher’s Note

All claims expressed in this article are solely those of the authors and do not necessarily represent those of their affiliated organizations, or those of the publisher, the editors and the reviewers. Any product that may be evaluated in this article, or claim that may be made by its manufacturer, is not guaranteed or endorsed by the publisher.

## Footnotes

1 The use of masked priming paradigms has also contributed to different patterns of findings, including monolingual orthographic priming studies that have manipulated neighborhood density and/or frequency in adults (e.g., Davis and Lupker, 2006; Nakayama et al., 2008; Massol et al., 2010) and in children (e.g., Castles et al., 2003; Tamura et al., 2017).

2 Although total orthographic neighborhood density was analyzed continuously in all models, it was dichotomized using a median split for the presentation of means to facilitate comparisons with standard analyses of variance (ANOVAs).
