# Supplementary material for: Eye Movement Measures of Within-Language and Cross-Language Activation During Reading in Monolingual and Bilingual Children and Adults: A Focus on Neighborhood Density Effects
Source: Front Psychol. 2021 Oct 27;12:674007. doi: 10.3389/fpsyg.2021.674007 (PMC8578698; doi:10.3389/fpsyg.2021.674007)
Supplement: Supplementary file 1 [file Data_Sheet_1.docx]

**Supplementary Appendix**

Table A1

| *Means and (standard deviations) of key lexical properties for all words in paragraphs.*   \| **All Words in Paragraphs** \| \| \| \| \| \| \| \| \| \| --- \| --- \| --- \| --- \| --- \| --- \| --- \| --- \| --- \| \| Lexical Property \| Paragraph 1 \| \| Paragraph 2 \| \| Paragraph 3 \| \| Paragraph 4 \| \| \| English \| French \| English \| French \| English \| French \| English \| French \| \| Length \| 4.10  (1.70) \| 4.57  (2.39) \| 4.25  (1.80) \| 4.91  (2.30) \| 4.11  (1.73) \| 4.74  (2.54) \| 4.54  (2.21) \| 4.80  (2.75) \| \| Frequency \| 5,588.34 (9,003.08) \| 4,327.02  (6,240.65) \| 5,883.99  (9,598.23) \| 3,091.29  (4,635.36) \| 6,117.25  (9,475.65) \| 4,669.57  (6,567.06) \| 4,506.35  (7,615.50) \| 4,460.23  (6,949.79) \| \| Predictability \| 0.23  (0.30) \| 0.23  (0.29) \| 0.33  (0.34) \| 0.34  (0.33) \| 0.27  (0.31) \| 0.26  (0.28) \| 0.28  (0.29) \| 0.29  (0.31) \| \| Mean Bigram Frequency \|  \|  \|  \|  \|  \|  \|  \|  \| \| Within-  Language \| 1,528.28  (767.85) \| 2,003.84  (1,070.40) \| 1,472.02  (731.06) \| 2,267.88  (1,215.95) \| 1,601.64  (777.40) \| 2,029.63  (1,066.49) \| 1,469.59  (726.95) \| 1,940.42  (1,018.50) \| \| Cross-  Language \| 1,523.26  (1,094.77) \| 1,474.56  (746.53) \| 1,457.21  (880.34) \| 1,704.07  (859.47) \| 1,662.61  (1,000.56) \| 1,551.61  (753.78) \| 1,564.39  (946.45) \| 1,481.00  (759.83) \| \| Total Phonological ND \|  \|  \|  \|  \|  \|  \|  \|  \| \| Within-  Language \| 20.07  (16.37) \| 38.36  (29.15) \| 21.06  (16.42) \| 40.40  (29.11) \| 20.24  (14.96) \| 38.52  (29.94) \| 22.41  (18.56) \| 35.41  (30.89) \| \| Cross-  Language \| 13.19  (22.47) \| 11.93  (12.24) \| 11.94  (24.95) \| 12.20  (13.20) \| 9.12  (16.47) \| 12.08  (13.75) \| 12.35  (22.59) \| 11.00  (13.35) \| \| Total Orthographic ND \|  \|  \|  \|  \|  \|  \|  \|  \| \| Within-  Language \| 10.93  (7.51) \| 8.03  (5.66) \| 9.89  (6.89) \| 8.00  (5.87) \| 11.24  (7.77) \| 7.37  (5.54) \| 10.17  (8.38) \| 7.64  (6.05) \| \| Cross-  Language \| 3.65  (4.65) \| 8.04  (7.91) \| 3.75  (4.36) \| 7.55  (7.82) \| 4.85  (5.25) \| 7.35  (8.11) \| 4.34  (5.10) \| 7.91  (8.23) \| \| Orthographic ND of HF Neighbors \|  \|  \|  \|  \|  \|  \|  \|  \| \| Within-  Language \| 1.13  (1.68) \| 1.39  (1.86) \| 1.02  (1.25) \| 1.25  (1.52) \| 1.52  (1.93) \| 1.02  (1.43) \| 0.81  (1.44) \| 1.19  (1.73) \| \| Cross-  Language \| 0.35  (0.95) \| 0.78  (1.54) \| 0.40  (0.77) \| 0.43  (0.77) \| 0.56  (1.35) \| 0.38  (0.91) \| 0.49  (1.24) \| 0.65  (1.42) \| \| Orthographic NF of HF Neighbors \|  \|  \|  \|  \|  \|  \|  \|  \| \| Within-  Language \| 8,961.25  (11,865.09) \| 9,128.46  (9,385.57) \| 8,851.24  (13,984.60) \| 8,898.47  (9,046.87) \| 9,502.24  (15,113.30) \| 9,633.66  (9,900.83) \| 8,077.74  (10,325.06) \| 8,731.24  (9,238.28) \| \| Cross-  Language \| 7,678.74  (6,390.81) \| 9,770.87  (10,643.92) \| 5,286.76  (6,408.96) \| 7,611.72  (10,004.31) \| 4,507.66  (5,568.15) \| 18,783.63  (13,104.47) \| 10,129.56  (7,402.98) \| 11,835.05  (13,444.18) \| |
| --- | --- | --- | --- | --- | --- | --- | --- | --- | --- | --- | --- | --- | --- | --- | --- | --- | --- | --- | --- | --- | --- | --- | --- | --- | --- | --- | --- | --- | --- | --- | --- | --- | --- | --- | --- | --- | --- | --- | --- | --- | --- | --- | --- | --- | --- | --- | --- | --- | --- | --- | --- | --- | --- | --- | --- | --- | --- | --- | --- | --- | --- | --- | --- | --- | --- | --- | --- | --- | --- | --- | --- | --- | --- | --- | --- | --- | --- | --- | --- | --- | --- | --- | --- | --- | --- | --- | --- | --- | --- | --- | --- | --- | --- | --- | --- | --- | --- | --- | --- | --- | --- | --- | --- | --- | --- | --- | --- | --- | --- | --- | --- | --- | --- | --- | --- | --- | --- | --- | --- | --- | --- | --- | --- | --- | --- | --- | --- | --- | --- | --- | --- | --- | --- | --- | --- | --- | --- | --- | --- | --- | --- | --- | --- | --- | --- | --- | --- | --- | --- | --- | --- | --- | --- | --- | --- | --- | --- | --- | --- | --- | --- | --- | --- | --- | --- | --- | --- | --- | --- | --- | --- | --- | --- | --- | --- | --- | --- | --- | --- | --- | --- | --- | --- | --- | --- | --- | --- | --- |

*Note: ND = neighborhood density; NF = neighborhood frequency; HF = higher-frequency.*

Table A2

| *Means and (standard deviations) of key lexical properties for target words in paragraphs.*   \| **Target Words in Paragraphs** \| \| \| \| \| \| \| \| \| \| --- \| --- \| --- \| --- \| --- \| --- \| --- \| --- \| --- \| \| Lexical Property \| Paragraph 1 \| \| Paragraph 2 \| \| Paragraph 3 \| \| Paragraph 4 \| \| \| English \| French \| English \| French \| English \| French \| English \| French \| \| Length \| 5.72  (1.59) \| 5.96  (1.72) \| 5.33  (1.37) \| 6.95  (1.88) \| 4.92  (1.20) \| 6.43  (2.02) \| 5.91  (1.75) \| 6.84  (2.47) \| \| Frequency \| 286.36  (468.53) \| 348.66  (723.62) \| 128.23  (181.22) \| 100.69  (224.78) \| 476.45  (802.46) \| 758.30  (1,797.35) \| 532.77  (1,038.03) \| 373.40  (805.26) \| \| Predictability \| 0.09  (0.18) \| 0.07  (0.10) \| 0.16  (0.20) \| 0.16  (0.24) \| 0.13  (0.25) \| 0.15  (0.22) \| 0.15  (0.24) \| 0.09  (0.16) \| \| Mean Bigram Frequency \|  \|  \|  \|  \|  \|  \|  \|  \| \| Within-  Language \| 1,679.56  (638.64) \| 2,363.78  (1,002.57) \| 1,622.00  (729.91) \| 2,946.78  (1,317.32) \| 1,765.26  (772.25) \| 2,328.61  (1,102.85) \| 1,726.13  (638.23) \| 2,403.79  (1,066.35) \| \| Cross-  Language \| 1,487.35  (708.83) \| 1,656.09  (580.39) \| 1,561.42  (968.37) \| 2,148.22  (819.09) \| 1,840.75  (1,024.59) \| 1,680.25  (747.54) \| 1,876.82  (1,012.58) \| 1,696.65  (735.02) \| \| Total  Phonological ND \|  \|  \|  \|  \|  \|  \|  \|  \| \| Within-  Language \| 13.08  (13.39) \| 29.23  (28.52) \| 12.17  (7.41) \| 18.70  (19.53) \| 16.12  (11.32) \| 31.86  (28.31) \| 12.77  (13.41) \| 19.39  (19.95) \| \| Cross-  Language \| 3.36  (8.71) \| 7.35  (13.08) \| 1.00  (3.46) \| 4.05  (8.61) \| 1.84  (5.42) \| 5.38  (10.49) \| 5.79  (13.84) \| 1.74  (5.23) \| \| Total  Orthographic ND \|  \|  \|  \|  \|  \|  \|  \|  \| \| Within-  Language \| 7.56  (8.11) \| 5.31  (5.09) \| 6.83  (4.55) \| 3.45  (3.69) \| 8.92  (6.99) \| 5.19  (4.91) \| 5.33  (4.97) \| 3.89  (3.73) \| \| Cross-  Language \| 0.48  (0.92) \| 3.62  (5.38) \| 2.25  (2.67) \| 1.00  (2.10) \| 3.12  (4.67) \| 1.29  (2.17) \| 1.05  (2.35) \| 1.55  (3.33) \| \| Orthographic ND of HF Neighbors \|  \|  \|  \|  \|  \|  \|  \|  \| \| Within-  Language \| 1.36  (2.34) \| 1.23  (1.42) \| 2.17  (2.21) \| 0.95  (1.50) \| 2.72  (2.81) \| 1.05  (1.69) \| 0.60  (1.33) \| 0.87  (1.71) \| \| Cross-  Language \| 0.12  (0.44) \| 0.38  (1.10) \| 0.42  (1.00) \| 0.25  (0.55) \| 1.24  (2.28) \| 0.05  (0.22) \| 0.19  (0.70) \| 0.37  (1.44) \| \| Orthographic NF of HF Neighbors \|  \|  \|  \|  \|  \|  \|  \|  \| \| Within-  Language \| 1,225.81  (1,992.03) \| 902.99  (2,062.78) \| 254.85  (345.82) \| 20.05  (25.80) \| 432.79  (486.08) \| 720.68  (604.34) \| 370.35  (763.88) \| 546.38  (983.56) \| \| Cross-  Language \| 1,927.45  (2,717.49) \| 411.30  (842.66) \| 2,743.13  (3,861.54) \| 41.39  (47.57) \| 373.16  (403.31) \| 224.16  (−)^1^ \| 2,973.50  (4,700.10) \| 1,199.05  (2282.17) \|   *Note: ND = neighborhood density; NF = neighborhood frequency; HF = higher-frequency.*  *^1^Only one target word in the French version of paragraph 3 had a higher-frequency cross-language orthographic neighbor.* |
| --- | --- | --- | --- | --- | --- | --- | --- | --- | --- | --- | --- | --- | --- | --- | --- | --- | --- | --- | --- | --- | --- | --- | --- | --- | --- | --- | --- | --- | --- | --- | --- | --- | --- | --- | --- | --- | --- | --- | --- | --- | --- | --- | --- | --- | --- | --- | --- | --- | --- | --- | --- | --- | --- | --- | --- | --- | --- | --- | --- | --- | --- | --- | --- | --- | --- | --- | --- | --- | --- | --- | --- | --- | --- | --- | --- | --- | --- | --- | --- | --- | --- | --- | --- | --- | --- | --- | --- | --- | --- | --- | --- | --- | --- | --- | --- | --- | --- | --- | --- | --- | --- | --- | --- | --- | --- | --- | --- | --- | --- | --- | --- | --- | --- | --- | --- | --- | --- | --- | --- | --- | --- | --- | --- | --- | --- | --- | --- | --- | --- | --- | --- | --- | --- | --- | --- | --- | --- | --- | --- | --- | --- | --- | --- | --- | --- | --- | --- | --- | --- | --- | --- | --- | --- | --- | --- | --- | --- | --- | --- | --- | --- | --- | --- | --- | --- | --- | --- | --- | --- | --- | --- | --- | --- | --- | --- | --- | --- | --- | --- | --- | --- | --- | --- | --- | --- | --- | --- | --- |

Table A3

*Effect sizes (β), standard errors (SE), t values, and p values for Model 1 – total within-language (L1) orthographic neighborhood density effects on L1 reading.*

*Note.* L1 = first-language; ND = neighborhood density; NF = neighborhood frequency; HF = higher-frequency; WIAT-II = Wechsler Individual Achievement Test – 2nd Edition; TONI-III = Test of Nonverbal Intelligence – 3rd Edition.

* *p* < .05; ** *p* < .01; *** *p* *<* .001

|  | **Gaze Duration** | | | | **Total Reading Time** | | | |
| --- | --- | --- | --- | --- | --- | --- | --- | --- |
| **Fixed Effects** | *β* | *SE* | *t* | *p* | *β* | *SE* | *t* | *p* |
| Age group | 62.69 | 18.08 | 3.47 | .001** | 55.74 | 33.68 | 1.66 | .100 |
| Language group | -20.95 | 18.49 | -1.13 | .259 | -45.05 | 34.33 | -1.31 | .192 |
| Total within-language ND | -7.85 | 5.98 | -1.55 | .122 | -15.64 | 8.22 | -1.90 | .057 |
| Age group × Language group | -41.37 | 28.20 | -1.47 | .145 | -92.12 | 52.47 | -1.76 | .082 |
| Age group × Total within-language ND | -10.63 | 6.01 | -1.77 | .077 | -36.48 | 9.73 | -3.75 | .000*** |
| Language group × Total within-language ND | 5.37 | 6.02 | 0.89 | .371 | 2.14 | 9.76 | 0.22 | .826 |
| Age group × Language group × Total within-language ND | 4.11 | 12.01 | 0.34 | .715 | 41.36 | 19.46 | 2.13 | .034* |
| **Control Predictors** | *β* | *SE* | *t* | *p* | *β* | *SE* | *t* | *p* |
| Word length | 11.37 | 6.43 | 1.77 | .077 | 66.85 | 10.13 | 6.60 | .000*** |
| Word frequency | -33.68 | 5.38 | -6.26 | .000*** | -16.86 | 8.65 | -1.95 | .051 |
| Word predictability | -7.83 | 3.16 | -2.48 | .013* | -17.35 | 5.00 | -3.47 | .001** |
| Mean within-language bigram frequency | 12.49 | 3.14 | 3.98 | .000*** | 29.68 | 5.00 | 5.93 | .000*** |
| Total within-language phonological ND | -1.40 | 4.33 | -0.32 | .747 | -0.59 | 6.97 | -0.09 | .932 |
| Within-language orthographic ND of HF neighbors | -1.28 | 3.71 | -0.34 | .731 | -36.02 | 6.00 | -6.00 | .000*** |
| Within-language orthographic NF of HF neighbors | 0.73 | 3.89 | 0.19 | .851 | 9.77 | 6.09 | 1.61 | .109 |
| L1 WIAT-II Word Reading | -27.56 | 7.32 | -3.76 | .000*** | -63.04 | 13.64 | -4.62 | .000*** |
| Current L1 exposure | -6.42 | 10.08 | -0.64 | .525 | -7.13 | 18.62 | -0.38 | .702 |
| TONI-III | 3.16 | 6.94 | 0.46 | .649 | 16.62 | 12.97 | 1.28 | .203 |
| Intercept | 283.93 | 10.63 | 26.71 | .000*** | 430.50 | 14.97 | 30.59 | .000*** |
| **Random Effects** | **Variance** | | | | **Variance** | | | |
|  | Intercept | | | | Intercept | | | |
| Participants | 3,808.86 | | | | 14,170.11 | | | |
| Paragraph version | 281.31 | | | | 157.12 | | | |
| Residual | 31,155.11 | | | | 81,564.28 | | | |

Table A4

*Effect sizes (β), standard errors (SE), t values, and p values for Model 2 – total within-language (L2) orthographic neighborhood density effects on L2 reading.*

|  | **Gaze Duration** | | | | **Total Reading Time** | | | |
| --- | --- | --- | --- | --- | --- | --- | --- | --- |
| **Fixed Effects** | *β* | *SE* | *t* | *p* | *β* | *SE* | *t* | *p* |
| Age group | 56.68 | 42.63 | 1.33 | .188 | 111.41 | 89.52 | 1.24 | .218 |
| Total within-language ND | -18.65 | 10.53 | -1.77 | .077 | -32.95 | 17.30 | -1.90 | .057 |
| Age group × Total within-language ND | -16.05 | 12.89 | -1.25 | .213 | -5.79 | 21.13 | -0.27 | .784 |
| **Control Predictors** | *β* | *SE* | *t* | *p* | *β* | *SE* | *t* | *p* |
| Word length | 25.85 | 12.50 | 2.07 | .039* | 20.34 | 20.43 | 1.00 | .320 |
| Word frequency | -13.20 | 13.01 | -1.02 | .311 | -61.91 | 21.58 | -2.87 | .004** |
| Word predictability | 2.19 | 8.03 | 0.27 | .785 | -1.81 | 13.23 | -0.14 | .891 |
| Mean within-language bigram frequency | -8.60 | 7.87 | -1.09 | .275 | 35.65 | 12.95 | 2.75 | .006** |
| Total within-language phonological ND | -21.22 | 11.16 | -1.90 | .059 | 19.27 | 18.68 | 1.03 | .303 |
| Within-language orthographic ND of HF neighbors | 10.31 | 7.64 | 1.35 | .178 | -0.22 | 12.50 | -0.02 | .986 |
| Within-language orthographic NF of HF neighbors | -13.07 | 8.02 | -1.63 | .104 | -27.67 | 13.15 | -2.11 | .035* |
| L2 WIAT-II Word Reading | -14.33 | 15.56 | -0.92 | .361 | -59.67 | 32.91 | -1.80 | .077 |
| Current L2 exposure | -7.59 | 19.10 | -0.40 | .692 | -23.04 | 40.17 | -0.57 | .568 |
| TONI-III | 11.85 | 17.21 | 0.69 | .494 | 18.64 | 36.52 | 0.51 | .611 |
| Intercept | 375.53 | 18.26 | 20.57 | .000*** | 606.57 | 37.32 | 16.25 | .000*** |
| **Random Effects** | **Variance** | | | | **Variance** | | | |
|  | Intercept | | | | Intercept | | | |
| Participants | 10,438.10 | | | | 52,041.23 | | | |
| Paragraph version | 308.17 | | | | 1,312.36 | | | |
| Residual | 61,943.24 | | | | 165,284.41 | | | |

*Note.* L2 = first-language; ND = neighborhood density; NF = neighborhood frequency; HF = higher-frequency; WIAT-II = Wechsler Individual Achievement Test – 2nd Edition; TONI-III = Test of Nonverbal Intelligence – 3rd Edition.

* *p* < .05; ** *p* < .01; *** *p* *<* .001

Table A5

*Effect sizes (β), standard errors (SE), t values, and p values for Model 3 – total cross-language (L2) orthographic neighborhood density effects on L1 reading.*

|  | **Gaze Duration** | | | | **Total Reading Time** | | | |
| --- | --- | --- | --- | --- | --- | --- | --- | --- |
| **Fixed Effects** | *β* | *SE* | *t* | *p* | *β* | *SE* | *t* | *p* |
| Age group | 45.67 | 21.72 | 2.10 | .037* | 44.94 | 44.07 | 1.02 | .309 |
| Language group | -12.37 | 22.46 | -0.55 | .582 | -49.55 | 45.46 | -1.09 | .277 |
| Total cross-language ND | -31.43 | 10.86 | -2.90 | .006** | -43.88 | 16.64 | -2.64 | .008** |
| Age group × Language group | -34.56 | 35.20 | -0.98 | .327 | -58.47 | 71.14 | -0.82 | .412 |
| Age group × Total cross-language ND | 1.38 | 5.44 | 0.25 | .800 | -7.33 | 10.51 | -0.70 | .486 |
| Language group × Total cross-language ND | -4.47 | 5.53 | -0.81 | .419 | -2.90 | 10.65 | -0.27 | .786 |
| Age group × Language group × Total cross-language ND | -9.42 | 10.87 | -0.87 | .387 | 1.03 | 21.00 | 0.05 | .961 |
| **Control Predictors** | *β* | *SE* | *t* | *p* | *β* | *SE* | *t* | *p* |
| Word length | 13.19 | 23.29 | 0.57 | .571 | 85.40 | 44.70 | 1.91 | .056 |
| Word frequency | 16.65 | 32.99 | 0.51 | .617 | 24.67 | 55.42 | 0.45 | .656 |
| Word predictability | 31.82 | 37.23 | 0.85 | .393 | 102.03 | 69.36 | 1.47 | .142 |
| Mean within-language bigram frequency | 68.89 | 39.11 | 1.76 | .086 | 106.46 | 64.74 | 1.64 | .100 |
| Mean cross-language bigram frequency | -43.43 | 47.54 | -0.91 | .367 | -76.05 | 79.15 | -0.96 | .337 |
| Total within-language phonological ND | -7.55 | 10.08 | -0.75 | .455 | -5.08 | 18.66 | -0.27 | .785 |
| Total cross-language phonological ND | -2.14 | 10.19 | -0.21 | .834 | 9.10 | 19.29 | 0.47 | .637 |
| Within-language orthographic ND of HF neighbors | -20.76 | 9.28 | -2.24 | .032* | -10.46 | 14.02 | -0.75 | .455 |
| Cross-language orthographic ND of HF neighbors | -46.49 | 15.44 | -3.01 | .004** | -79.28 | 24.69 | -3.21 | .001** |
| Within-language orthographic NF of HF neighbors | 9.66 | 18.16 | 0.53 | .596 | 47.56 | 31.43 | 1.51 | .131 |
| Cross-language orthographic NF of HF neighbors | -57.79 | 23.53 | -2.46 | .040* | -48.67 | 32.06 | -1.52 | .129 |
| L1 WIAT-II Word Reading | -33.87 | 8.18 | -4.14 | .000*** | -70.97 | 16.76 | -4.24 | .000*** |
| Current L1 exposure | 0.21 | 12.05 | 0.02 | .986 | 3.52 | 24.24 | 0.15 | .885 |
| TONI-III | 6.50 | 7.59 | 0.86 | .393 | 13.47 | 15.58 | 0.86 | .389 |
| Intercept | 341.23 | 31.91 | 10.69 | .000*** | 491.38 | 57.84 | 8.50 | .000*** |
| **Random Effects** | **Variance** | | | | **Variance** | | | |
|  | Intercept | | | | Intercept | | | |
| Participants | 3,178.15 | | | | 14,656.12 | | | |
| Paragraph version | 554.22 | | | | 1,434.79 | | | |
| Residual | 19,498.91 | | | | 72,638.26 | | | |

*Note.* L1 = first-language; L2 = second-language; ND = neighborhood density; NF = neighborhood frequency; HF = higher-frequency; WIAT-II = Wechsler Individual Achievement Test – 2nd Edition; TONI-III = Test of Nonverbal Intelligence – 3rd Edition.

* *p* < .05; ** *p* < .01; *** *p* *<* .001

Table A6

*Effect sizes (β), standard errors (SE), t values, and p values for Model 4 – total cross-language (L1) orthographic neighborhood density effects on L2 reading.*

|  | **Gaze Duration** | | | | **Total Reading Time** | | | |
| --- | --- | --- | --- | --- | --- | --- | --- | --- |
| **Fixed Effects** | *Β* | *SE* | *t* | *p* | *β* | *SE* | *t* | *p* |
| Age group | 87.71 | 73.43 | 1.20 | .237 | 122.96 | 114.66 | 1.07 | .287 |
| Total cross-language ND | -144.19 | 65.34 | -2.21 | .032* | -110.47 | 99.01 | -1.12 | .267 |
| Age group × Total cross-language ND | -13.88 | 18.78 | -0.74 | .460 | -22.34 | 30.82 | -0.73 | .469 |
| **Control Predictors** | *Β* | *SE* | *t* | *p* | *β* | *SE* | *t* | *p* |
| Word length | 23.72 | 54.14 | 0.44 | .675 | 29.60 | 68.61 | 0.43 | .681 |
| Word frequency | -50.16 | 81.04 | -0.62 | .555 | -262.38 | 107.75 | -2.44 | .026* |
| Word predictability | -86.39 | 79.54 | -1.09 | .313 | -50.96 | 95.18 | -0.54 | .640 |
| Mean within-language bigram frequency | 27.36 | 93.44 | 0.29 | .777 | 288.03 | 117.27 | 2.46 | .053 |
| Mean cross-language bigram frequency | -99.84 | 154.17 | -0.65 | .530 | -403.51 | 198.14 | -2.04 | .088 |
| Total within-language phonological ND | -65.48 | 75.31 | -0.87 | .425 | 5.00 | 88.28 | 0.06 | .958 |
| Total cross-language phonological ND | -21.52 | 42.92 | -0.50 | .651 | 47.95 | 45.16 | 1.06 | .411 |
| Within-language orthographic ND of HF neighbors | 31.40 | 23.92 | 1.31 | .216 | -2.24 | 31.03 | -0.07 | .944 |
| Cross-language orthographic ND of HF neighbors | -81.24 | 33.09 | -2.46 | .018* | -54.89 | 47.34 | -1.16 | .267 |
| Within-language orthographic NF of HF neighbors | -71.49 | 48.75 | -1.47 | .197 | -25.62 | 62.74 | -0.41 | .689 |
| Cross-language orthographic NF of HF neighbors | 14.02 | 31.39 | 0.45 | .658 | 25.60 | 46.79 | 0.55 | .586 |
| L2 WIAT-II Word Reading | 8.71 | 25.95 | 0.34 | .738 | -43.63 | 40.64 | -1.07 | .287 |
| Current L2 exposure | -30.02 | 31.93 | -0.94 | .351 | -54.62 | 49.67 | -1.10 | .276 |
| TONI-III | 17.56 | 29.26 | 0.60 | .551 | 19.29 | 44.94 | 0.43 | .670 |
| Intercept | 372.11 | 117.13 | 3.18 | .081 | 597.75 | 123.79 | 4.83 | .022* |
| **Random Effects** | **Variance** | | | | **Variance** | | | |
|  | Intercept | | | | Intercept | | | |
| Participants | 23,266.15 | | | | 54,017.23 | | | |
| Paragraph version | 15,608.12 | | | | 3,314.57 | | | |
| Residual | 66,612.26 | | | | 179,444.42 | | | |

*Note.* L1 = first-language; L2 = second-language; ND = neighborhood density; NF = neighborhood frequency; HF = higher-frequency; WIAT-II = Wechsler Individual Achievement Test – 2nd Edition; TONI-III = Test of Nonverbal Intelligence – 3rd Edition.

* *p* < .05; ** *p* < .01; *** *p* *<* .001
